# A soft decoding algorithm and hardware implementation for the visual prosthesis based on high order soft demodulation

**DOI:** 10.1186/s12938-016-0229-3

**Published:** 2016-09-26

**Authors:** Yuan Yang, Nannan Quan, Jingjing Bu, Xueping Li, Ningmei Yu

**Affiliations:** Department of Electronic Engineering, Xi’an University of Technology, Jinhua Road No. 5, Xi’an, 710048 China

**Keywords:** Differential amplitude phase-shift keying, Reed–Solomon code, Bit error rate, Visual prosthesis

## Abstract

**Background:**

High order modulation and demodulation technology can solve the frequency requirement between the wireless energy transmission and data communication. In order to achieve reliable wireless data communication based on high order modulation technology for visual prosthesis, this work proposed a Reed–Solomon (RS) error correcting code (ECC) circuit on the basis of differential amplitude and phase shift keying (DAPSK) soft demodulation. Firstly, recognizing the weakness of the traditional DAPSK soft demodulation algorithm based on division that is complex for hardware implementation, an improved phase soft demodulation algorithm for visual prosthesis to reduce the hardware complexity is put forward. Based on this new algorithm, an improved RS soft decoding method is hence proposed. In this new decoding method, the combination of Chase algorithm and hard decoding algorithms is used to achieve soft decoding. In order to meet the requirements of implantable visual prosthesis, the method to calculate reliability of symbol-level based on multiplication of bit reliability is derived, which reduces the testing vectors number of Chase algorithm. The proposed algorithms are verified by MATLAB simulation and FPGA experimental results. During MATLAB simulation, the biological channel attenuation property model is added into the ECC circuit.

**Results:**

The data rate is 8 Mbps in the MATLAB simulation and FPGA experiments. MATLAB simulation results show that the improved phase soft demodulation algorithm proposed in this paper saves hardware resources without losing bit error rate (BER) performance. Compared with the traditional demodulation circuit, the coding gain of the ECC circuit has been improved by about 3 dB under the same BER of $10^{-6}$. The FPGA experimental results show that under the condition of data demodulation error with wireless coils 3 cm away, the system can correct it. The greater the distance, the higher the BER. Then we use a bit error rate analyzer to measure BER of the demodulation circuit and the RS ECC circuit with different distance of two coils. And the experimental results show that the RS ECC circuit has about an order of magnitude lower BER than the demodulation circuit when under the same coils distance. Therefore, the RS ECC circuit has more higher reliability of the communication in the system.

**Conclusions:**

The improved phase soft demodulation algorithm and soft decoding algorithm proposed in this paper enables data communication that is more reliable than other demodulation system, which also provide a significant reference for further study to the visual prosthesis system.

## Background

**Fig. 1:**
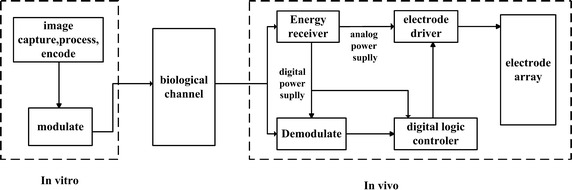
Schematic of visual prosthesis. This * figure* is a block diagram of the visual prosthesis system, including the external one and the implanted one

Visual prosthesis is usually composed of external and internal parts, as illustrated in Fig. 1. Since we study the communication error performance in vitro and vivo, the modulation, biological channel and demodulation modules are the significant parts that we concentrate on in this paper.

High-performance implantable biomedical microsystems mostly take advantage of a wireless interface to communication between the implanted modules to the external controller. Current-generation cortical (and retinal) visual prostheses are being researched to transfer energy and data with wireless way. The maximum carrier frequency for biomedical implants is limited to a few tens of megahertz due to the self-resonance frequency of the coupled coils, the energy loss in the transmission circuit, and energy dissipation in the tissue [1–4]. The significant consideration of the frequency limitation for wireless power transfer comes from the absorption of electromagnetic energy by tissues, which increases exponentially with frequency [5]. However, data transmission requires a data frequency high enough to stimulate electrodes so that the information can be received in real-time without distortion. So the trade-off of carrier frequency requirement is obvious between the power transmission and the data communication [6, 7]. Some scholars adopt two pairs of coils to transfer energy and data with their own frequency respectively, but this way will increase the area of the circuit [8]. In order to reduce the secondary implant volume, some researchers put forward two orthogonal coils assembly structure for energy and data transmission respectively, but there is the mutual interference between coils assembly [9].

Considering the safety of the implantable devices, we adopt a pair of coils to transfer date and energy with the same carrier frequency. Thus it is important to select an appropriate digital modulation and demodulation schemes and a suitable carrier frequency to meet the requirements of energy efficient and data rate. Since the energy transmission must adopt low carrier frequency, data transmission requires a high carrier frequency. With high order modulation and demodulation, a lower frequency of carrier can achieve a high rate of data transmission. Then a trade-off can be made between the energy transmission and the data communication in visual prosthesis system [10–12].

Nevertheless, When the data rate is high enough, it will appear some error bits. In order to achieve the reliability of the data communication, ECC circuit is needed. Literature [1] used Hamming code, but only one-bit error that occurs in the 24-bit unit can be corrected. Therefore, ECC methods for visual prosthesis are studied in this paper and RS code is selected. On one hand, RS code is a non-binary coding system that fits high order modulation and demodulation. What’s more, as a widely used coding system in wireless communication, the error correction ability of RS code is strong, both the random errors and burst errors can be corrected effectively. On the other hand, soft demodulation and soft decode can further improve the quality of communication [1, 13–16]. Hence RS code and DAPSK soft modulation and demodulation are combined to improve the reliability of the visual prosthesis system communication in this paper. Meanwhile, the algorithms are modified so to simplify hardware implementation.

## Methods

### Modulation parameters selection for visual prosthesis

As mentioned above, in this work data and energy would be transmitted using single pair of coils with the same carrier frequency. In order to meet the requirements of energy efficiency, the carrier frequency should not be too high to be absorbed by the tissues, and according to [17] 2 MHz is selected for the carrier frequency in our scheme. And to obtain high data rate at the same time, we take 16DAPSK method as a sample of high order data modulation in this work, which composed of 2DASK and 8DPSK, each symbol of modulated signal contains 4-bit information. At a symbol rate of 2 MHz, the data rate is 8 Mbps. Compared with the traditional methods like BPSK (binary phase shift keying) and ASK (amplitude shift keying), this way can improve the data rate at least four times higher, and what’s more, the data and energy can be transmitted with single pair of coils. Meanwhile, in terms of the experiment we did before, with the distance of 2 cm between the primary coil and the secondary coil, the fresh pigskin on the day to encircle the secondary coil to imitate the tissue of eyes. Then using a spectrum analyzer, we concluded that the transfer efficiency is $77\,\%$ with a transmitting terminal power of 285 mW and a receiving terminal power of 219 mW in 16DAPSK circuit [14].

### Demodulation principle

Each symbol consists of 4 bits in 16DAPSK, that is $S_{i}=\{A,B,C,D\}$. The A is used for amplitude modulation. BCD is used for phase modulation. So soft demodulation is carried out bit by bit, the A, B, C and D.

#### Soft demodulation of 16DAPSK [18]

*Soft output of differential amplitude* The A (differential amplitude) can be obtained by $\gamma _{i,k}$. The $\gamma _{i,k}$ distributing in the 1/*a*, 1, a. That is near the 0.5, 1, 2 when $a=2$. The soft output is transforming $\gamma _{i,k}$ as following:

1 $$\begin{aligned} If \;\; \gamma _{i,k}<1,\; D3=4\gamma _{i,k}-3{:}\quad Else\quad D3=-2\gamma _{i,k}+3 \end{aligned}$$

After the transformation, $\gamma _{i,k}$ will distribute in the −1, 1, −1. If D3 is less than 0, the value of A is judged to be 1; Else, A is judged to be 0. The absolute value of D3 is the size of the reliability that A is judged to be 0 or 1. Therefore, the D3 is taken as the soft output of the differential amplitude.

**Fig. 2:**
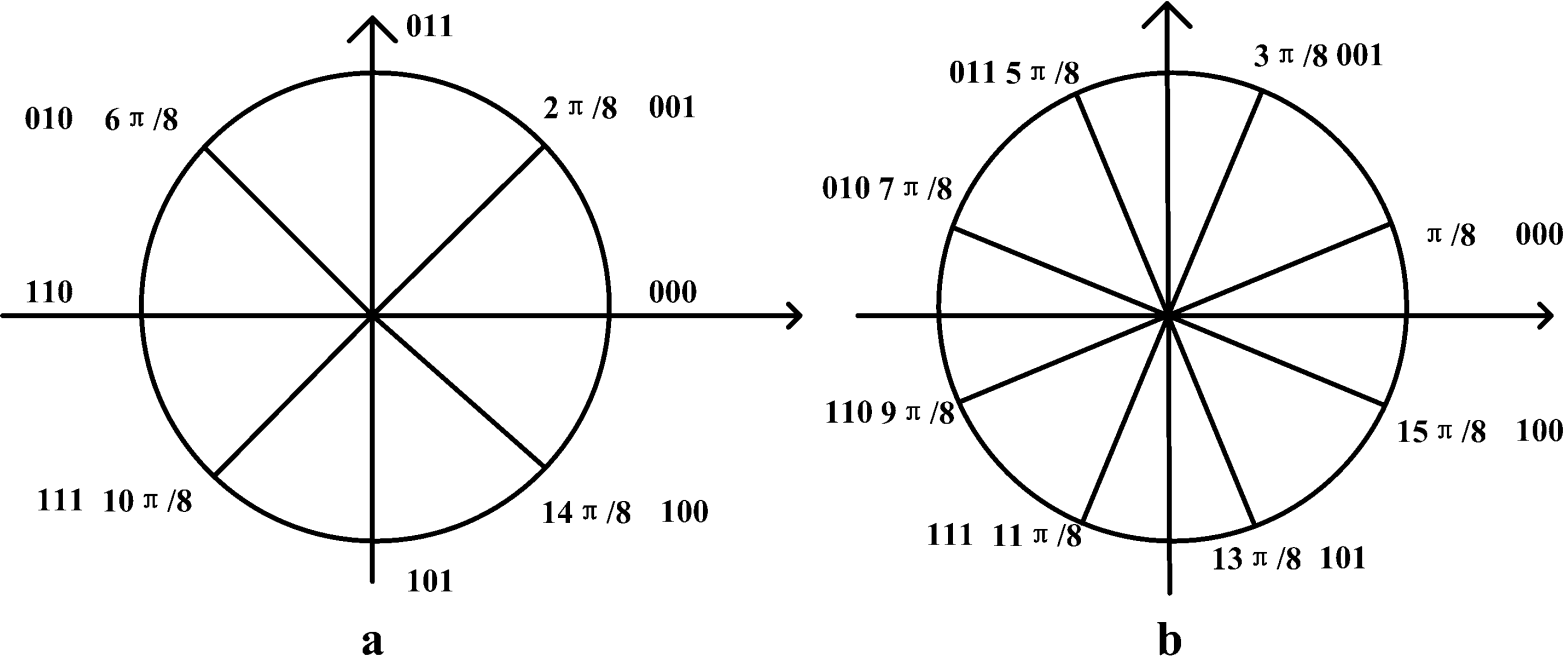
Signal space. **a** The signal space diagram of phase coded for BCD three bits; **b** rotate the received differential complex vector counterclockwise $\pi /8$

*Phase soft demodulation* Figure 2a is the signal space diagram of phase coded for BCD three bits. When the BCD takes 001, the differential phase $\Delta \phi _{i,k}$ is $2\pi /8.$

Rotate the received differential complex vector counterclockwise $\pi /8$ as shown in Fig. 2b. It can be seen that D is symmetrical to both X and Y axis, soft demodulation output of the first path can be obtained by the following formula:

2 $$\begin{aligned} D_{i,k,0}=-\pi /8\arg [|\cos (\Delta \phi _{i,k}+\pi /8)|+j|\sin (\Delta \phi _{i,k}+\pi /8)|]+2 \end{aligned}$$

The soft output of second bit C is:

3 $$\begin{aligned} D_{i,k,1}=\cos (\Delta \phi _{i,k}+\pi /8)/\cos (3\pi /8) \end{aligned}$$

After rotating the received differential complex vector clockwise $3\pi /8$, the soft output of third bit B can be got as following:

4 $$\begin{aligned} D_{i,k,2}=\cos (\Delta \phi _{i,k}-3\pi /8)/\cos (3\pi /8) \end{aligned}$$

#### Improved phase soft demodulation

As above, the phase soft demodulation involves argument arithmetic and division operation of trigonometric. The hardware implementation that supports these operations is complex. Therefore, the above method is improved in this paper for a more feasible hardware solution. According to the signal space shown in Fig. 2, soft demodulation can be done bit by bit as following:

5 $$\begin{aligned} If\;\; |\cos (\Delta \phi _{i,k})|>0.707, \quad D_{0}=3.4|\cos (\Delta \phi _{i,k})|-2.4 \end{aligned}$$

6 $$\begin{aligned} If\;\; |\cos (\Delta \phi _{i,k})|<0.707, \quad D_{0}=1.4|\cos (\Delta \phi _{i,k})|-1 \end{aligned}$$

If $D_{0}$ is less than 0, the value of D will be 1; else D is judged to be 0. The absolute value of $D_{0}$ is the size of the reliability for $D_{0}$ is judged to be 0 or 1.

$D_{1}$ the second bit soft output of the demodulator can be obtained as the following: The C is symmetry about X axis, because the triangle function $\cos$ value in the first and fourth quadrant is positive, and in the second and third quadrant is negative, so $D_{1}=\cos (\Delta \phi _{i,k})$ can be used as the second soft output.

$D_{2}$ the third bit soft output of the demodulator can be determined as the following: It can be seen that B is 0 in the first half circle, and in the second half it is 1, so $D_{2}=\sin (\Delta \phi _{i,k})$ can be used as the third soft output.

In the improved algorithm, the soft output reliability size of differential phase is from 0 to 1, which is consistent with the soft output of the differential amplitude. This is the foundation of the accuracy of reliability sorting. What’s more, it directly uses $\cos (\Delta \phi _{i,k})$ and $\sin (\Delta \phi _{i,k})$ as soft demodulation output without increasing additional circuit, to simplify the implementation circuit and save resources.

### RS decoding

#### Improved calculation of symbol level reliability

The fast RS soft decoding based on symbol level is adopted, and using the low reliability symbols to generate the test vectors in this paper. So it is necessary to compute the reliability of symbols. But not all the low reliability symbols are error symbols, thus selecting the appropriate symbol reliability algorithm to improve the accuracy of symbol reliability is especially important [19, 20].

For RS (15,9) error correcting codes in BPSK modulation and demodulation, each symbol consists of 4 bits, that is $R_{i}=\{A,B,C,D\}$, and the modulation and demodulation method for each bit is uniform, so the minimum reliability of 4 bits can be used as the symbol reliability, that is $L_{i}=\min \{|A|,|B|,|C|,|D|\}$.

However, in 16DAPSK, soft demodulation is bit by bit. Five different symbol reliability algorithms are constructed in this paper, such as 4  bits reliability addition algorithm, minimum bit reliability algorithm, multiplication of bit reliability algorithm, amplitude reliability algorithm, and phase reliability algorithm. The formulas are listed as following:

7 $$\begin{aligned} L_{i}=|A|+|B|+|C|+|D| \end{aligned}$$

8 $$\begin{aligned} L_{i}=\min \{|A|,|B|,|C|,|D|\} \end{aligned}$$

9 $$\begin{aligned} L_{i}=|A|*|B|*|C|*|D| \end{aligned}$$

10 $$\begin{aligned} L_{i}=|A| \end{aligned}$$

11 $$\begin{aligned} L_{i}=|B|+|C|+|D| \end{aligned}$$

If the transmitted symbol is $C=\{C_{14},C_{13},\ldots ,C_{1},C_{0}\}$, the symbols demodulator received is $H=\{H_{14},H_{13},\ldots ,H_{1},H_{0}\}$ by hard decision in a certain Eb/No(bit signal to noise ratio). Assuming that the subscript of the three symbols with smallest reliability respectively are m0, m1, and m2. If the total error bit number on an encoding word ($H_{i} \ne C_{i}$ is an error symbol) is N, and N3 is the number of $H_{m0} \ne C_{m0}$, $H_{m1} \ne C_{m1}$, $H_{m2} \ne C_{m2}$. So, N3/N is the representation of the reliability accuracy. That means closer to 1 the N3/N is, the higher accuracy of the reliability will be. From the simulation of the five symbol reliability algorithms, the multiplication of bit reliability algorithm is selected finally.

#### The generation of test vector

Chase algorithm (a class of iterative decoding algorithm proposed by Chase in 1972) establishes test vectors according to the symbol reliability of received vector, test vectors generates candidate set of codes after the hard decoder, from the candidate codes the optimal code will be selected as the decoding output [21, 22].

For binary code: choose $\lfloor d_{min}/2 \rfloor$ symbols with minimum reliability to establish test vectors. One symbol is 1 bit. So test vectors number is $2^{d_{min}/2}$ in total.

For RS (15,9), $d_{min}/2=3$, each symbol has 4  bits, so the number of test vector is $16^3$. But the complexity of hardware circuit increases with the number of possible vectors. To meet the requirements of the implantable visual prosthesis, two  bits of a symbol with low reliability are used to generate test vectors. So test vectors number is $4^{3}=64$, leading to the reduced complexity of the circuit which is more suitable for implantation.

## Results

### MATLAB modeling and simulation

**Fig. 3:**
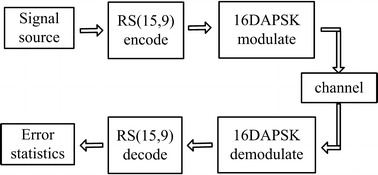
Block diagram of the ECC circuit, which consists of seven parts

Figure 3 is a block diagram of the ECC circuit, mainly include a pseudo-random binary sequence generation module, a RS encoding module, a modulation module, a demodulation module, a RS decoding module, a channel module and an error statistics module.

**Fig. 4:**
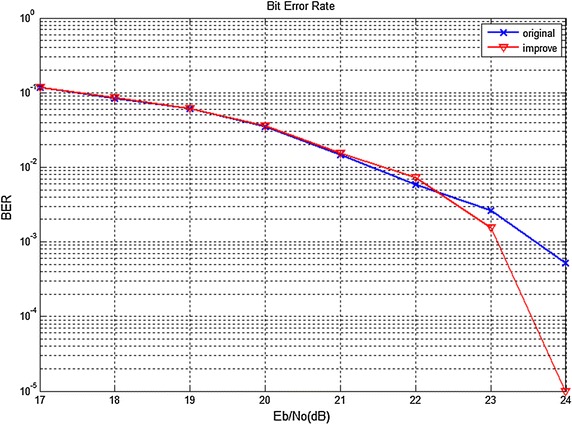
The error performance comparison of the two phase soft demodulation algorithms. This figure is the BER simulation result of the original phase soft demodulation algorithm and the improved phase soft demodulation algorithm under different Eb/No

The two curves on Fig. 4 are the BER under different Eb/No of the phase soft demodulation algorithm and the improved phase soft demodulation algorithm of the demodulation scheme respectively. There is no argument arithmetic and division operation of trigonometric in the later one. It can be seen that when Eb/No is lower than 23 dB the two algorithms possess similar BER. But for Eb/No above 23 dB, the improved phase soft demodulation algorithm can achieve lower BER. As a result, the improved phase soft demodulation algorithm can reduce the hardware resources without loss of BER performance.

**Fig. 5:**
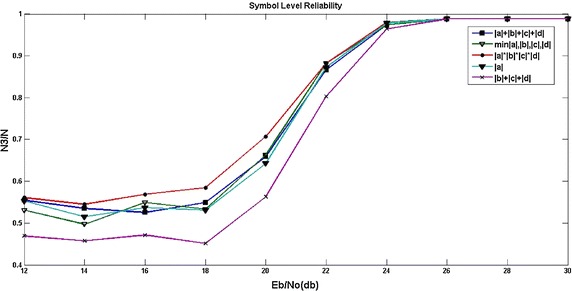
The simulation result of symbol level reliability with five different methods

Figure 5 is the simulation result of symbol level reliability with five different methods mentioned above. It can be seen that the marked error symbol by reliability is not the actual error symbol under low Eb/No. With the improvement of Eb/No, the accuracy of reliability is also improved and ultimately the accuracy reaches to 1, which means finally all the marked error symbols are error symbols. From the figure, it is also observed that the method of bit multiplication reliability calculation has the highest accuracy. Therefore, the multiplication of bit reliability method with high accuracy is adopted in this paper.

**Fig. 6:**
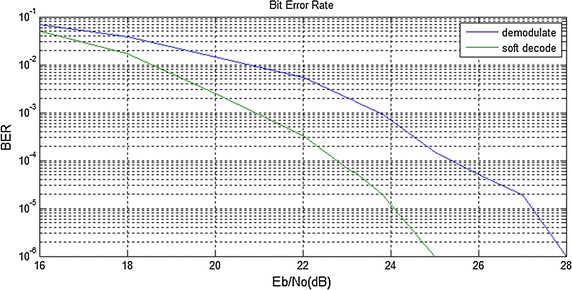
The error performance comparison of the demodulation circuit and the RS ECC circuit. This figure is the BER simulation result of the demodulation signal and the error correcting output signal when compared with the signal source respectively

Two curves on Fig. 6 are the BER of the demodulation signal and the error correcting output signal respectively. Considering the signal transmission in the biological channel with attenuation, in our previous work we built and analyzed the heterogeneous biological channel model. According to the analysis and experimental results, when the electromagnetic wave goes through the heterogeneous channel with 1 mm skin, 4 mm fat, 13 mm muscle, the attenuation would be $17\,\%$. This characteristics model is added to the RS ECC circuit for visual prosthesis. The MATLAB simulation results show that when the Eb/No is more than 18 dB, the BER gap is gradually increasing between demodulation signal and ECC signal, it reached an order of magnitude when Eb/No is about 21 dB. Under the same BER of ${10^{-6}}$, the coding gain improved by about 3 dB.

### Experimental verification

**Fig. 7:**
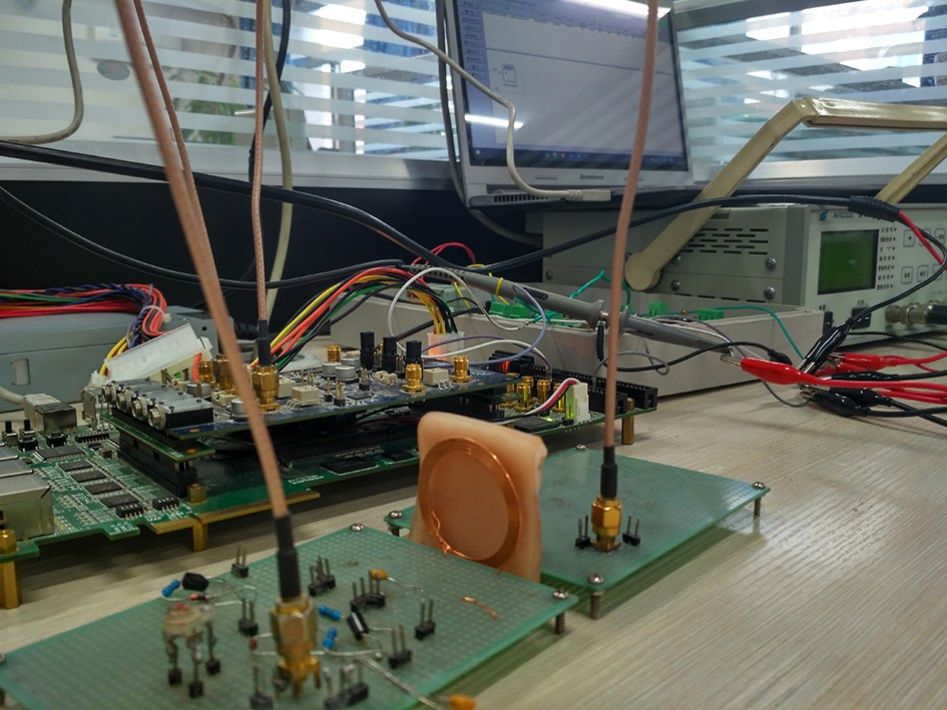
Verification platform. This* figure* is the wireless data transmission processing experiment platform

The design is verified by the wireless data transmission processing experiment platform, set up by our project team as shown in Fig. 7. The experimental platform is composed of the power and data transceiver coils and the secondary coil is encircled by the fresh pigskin to imitate the tissue of eyes, the Altera Stratix IV GX series of EP4SGX530KH40C2 development board, a high-speed AD/DA transfer card and a bit error rate analyzer is also added. And we use PRBS15 generated by the bit error rate Analyzer in our experiments. The effect of the coils coupling can reach at most $88.01\,\%$ [23].

In the experiment, the input signal is processed by coding circuit and DAPSK modulation circuit. And then the modulated digital signal is converted to analog signal by the AD/DA card. The sampling frequency is 16 MHz and the bit width is 14, then it is sent out by primary coil. The signal received by the receiving coil is then converted into 14 bits digital signal. The digital signal eventually enters the FPGA for demodulating and decoding. The demodulation system shows that the delay of the output is less than 0.04 ms, the data can be transmitted in real-time.

The verification schemes of 16DAPSK soft demodulation circuit were carried out under different coils distances. Due to the coils coupling transmission attenuation, the magnitude of transmission attenuation increases with the distance of coils. So, with the coils distance increases, the demodulator output will generate error bits. To verify the ECC circuit, the coils can be adjusted to the distance under which the demodulator outputs error bits. If the output of the ECC circuit is still consistent with the input data, it can be concluded that the error correction circuit is successful.

**Fig. 8:**
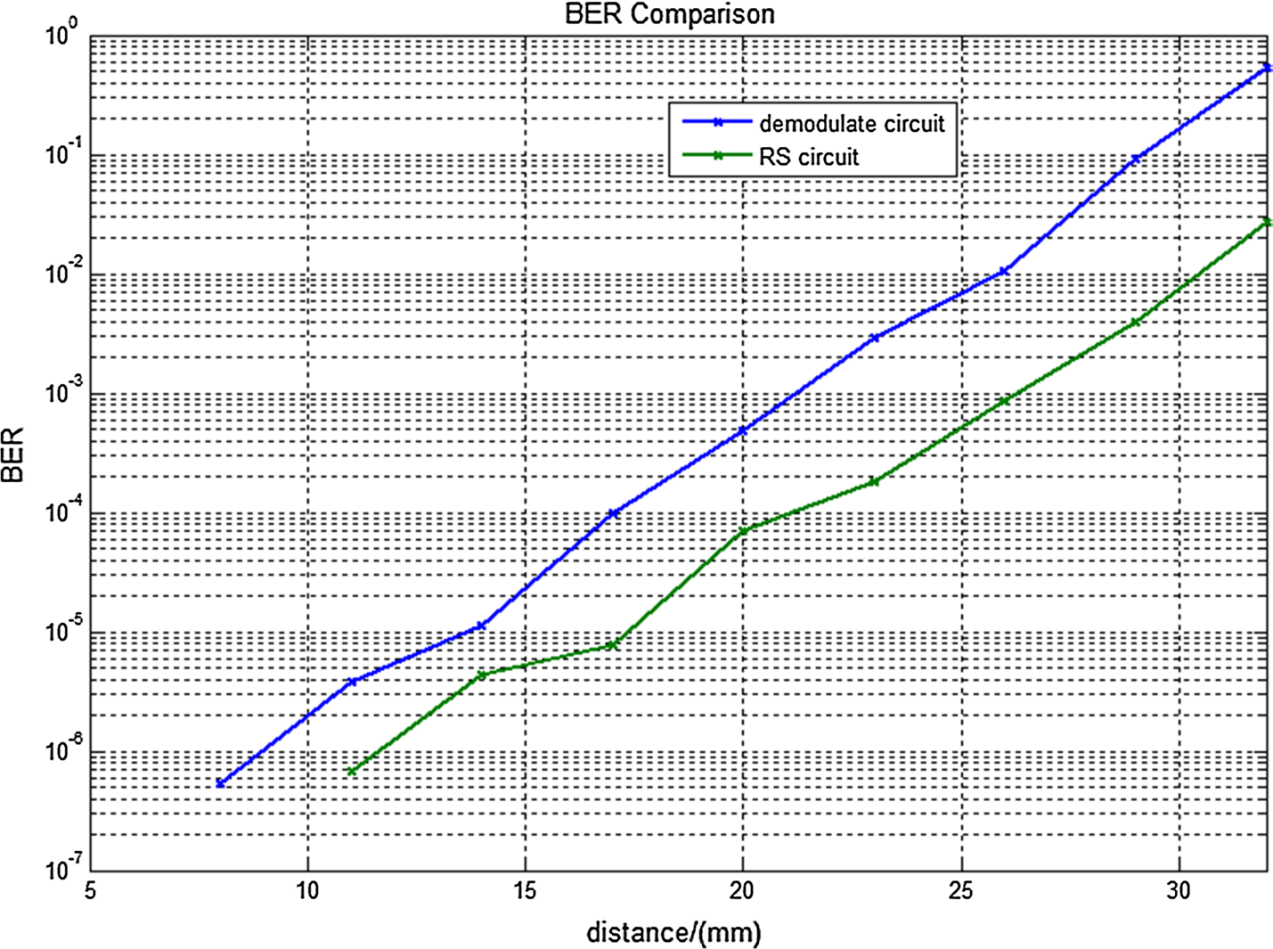
Experimental results of the demodulation circuit and the RS ECC circuit. This* figure* is the BER under different distance of two coils of the demodulation circuit and the RS ECC circuit measured by a bit error rate analyzer

Figure 8 is the BER under different distance of two coils with and without ECC circuit. When the two coils distance is very close, BER is almost zero. Although the BER is higher and higher with the increase of coils spacing, the ECC circuit take the lower BER. Therefore the proposed new circuit can improved the reliability of communication.

**Fig. 9:**
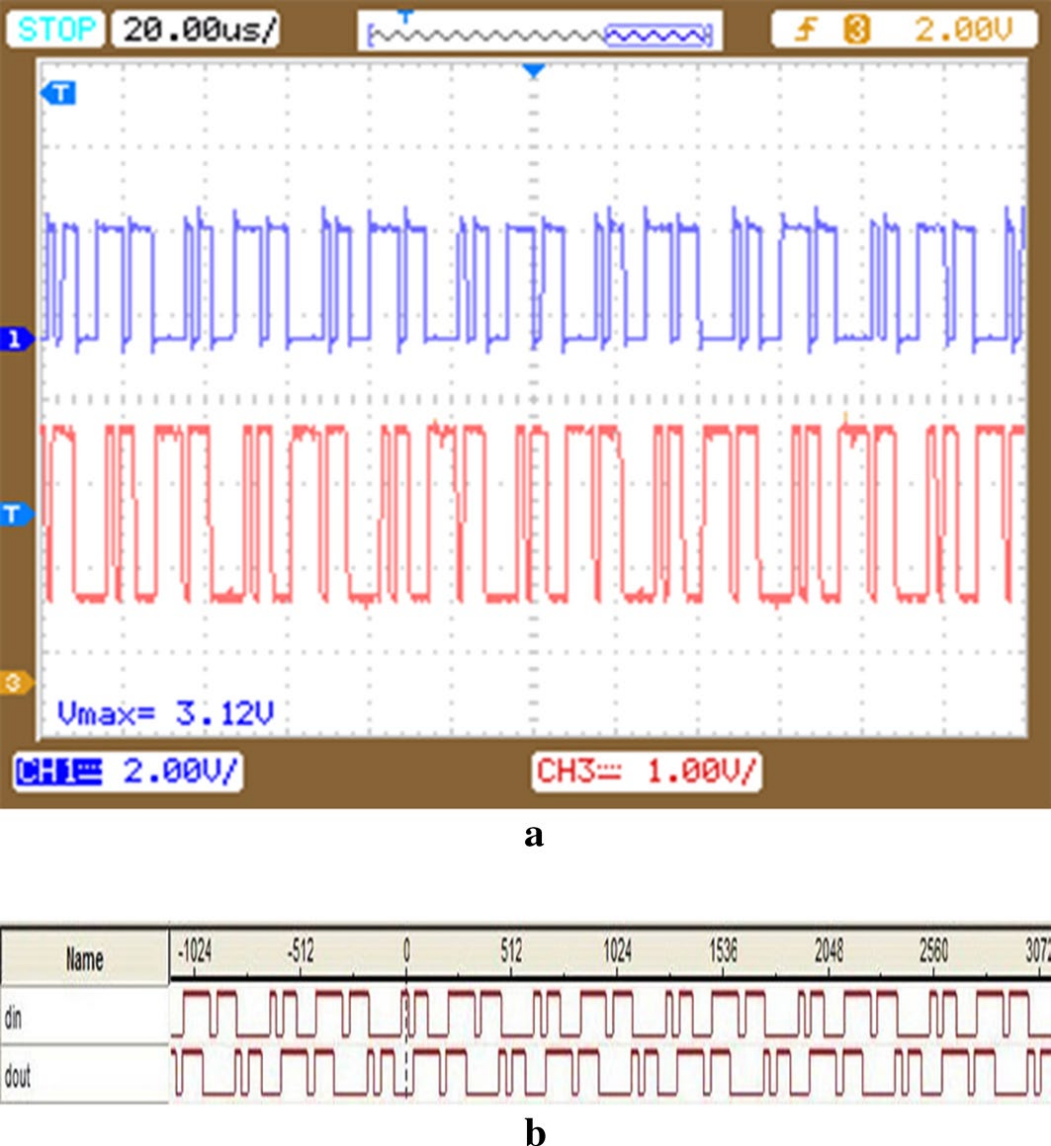
DAPSK circuit experiment of coils distance of 2 cm. **a** The oscilloscope result of the soft demodulation with the coils distance of 2 cm; **b** the logic analyzer result of the soft demodulation with the coils distance of 2 cm

Figure 9 is the oscilloscope and logic analyzer results of the soft demodulation with the coils distance of 2 cm. The first line is the input signal, and the second line is the output signal of soft demodulation in Fig. 9a. It can be seen that there is a certain delay between the input and output of the demodulator in Fig. 9, but the output is still consistent with the input. This indicates that the modulation and demodulation circuit are designed correctly.

**Fig. 10:**
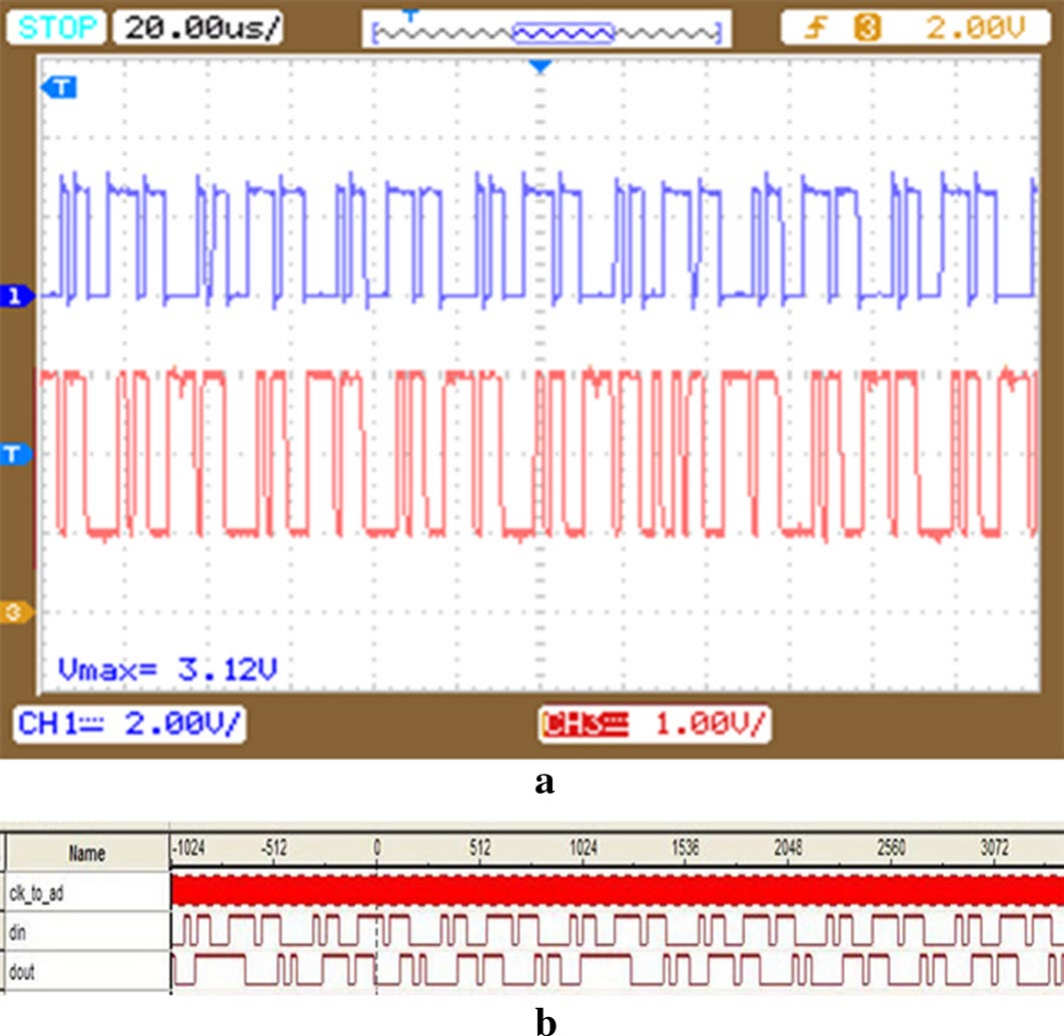
Soft demodulation result of coils distance of 3 cm. **a** The oscilloscope result of the soft demodulation with the coils distance of 3 cm; **b** the logic analyzer result of the soft demodulation with the coils distance of 3 cm

Figure 10 is the oscilloscope and logic analyzer result of the soft demodulation with the coils distance of 3 cm. It can be observed that the demodulation output appears erroneous.

**Fig. 11:**
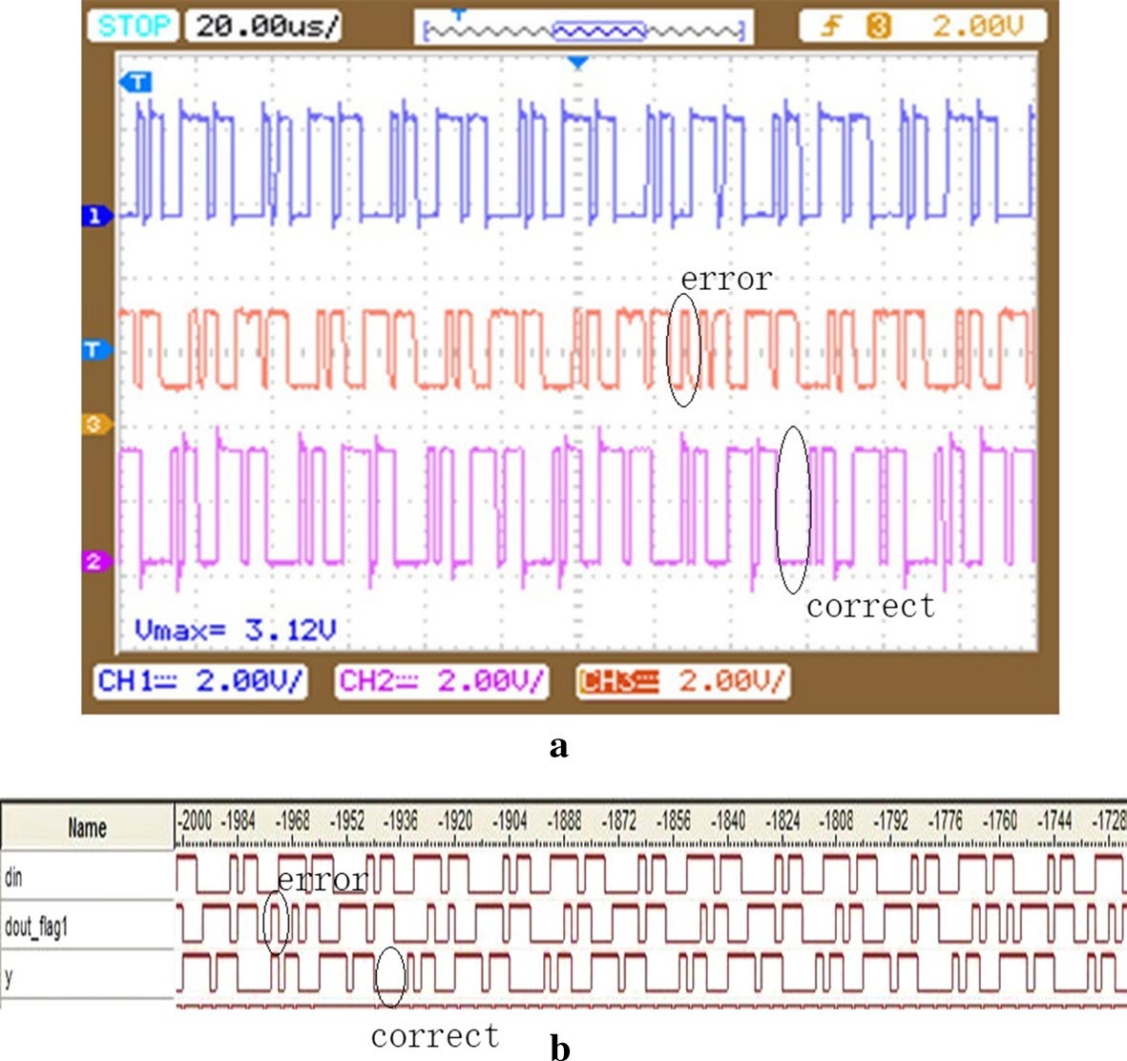
RS circuit experiment of coils distance of 3 cm. **a** The oscilloscope result of the experiments that applies error correction code to the condition of 3 cm coils distance; **b** the logic analyzer result of the experiments that applies error correction code to the condition of 3 cm coils distance

Figure 11 is the result of the experiments that applies ECC circuit to the condition of 3 cm coils distance. The first column in Fig. 11a is the input signal, the second column is the result of the soft demodulation, and the third one is the output signal of the ECC circuit. From the Fig. 11 it can be concluded that the output of the soft demodulation appears errors, whereas the output of the ECC circuit is consistent with the input. That is to say, the error correction circuit is capable of correcting error bits.

As for the implants side, compared with the traditional demodulation circuit, the power-consumption of the coding technique increases from 1.51 to 1.89 mW, and the RS coding technique would occupy $9.34\,\%$ more area.

## Conclusions

The improved 16DAPSK phase soft demodulation algorithm requires low hardware complexity and is, therefore, conducive to hardware implementation. Through the simulation of BER, it can be seen that the modified algorithm does not bring the loss of BER performance. Experiments were carried out to further verify the correctness of the soft demodulation algorithm. MATLAB simulation results show that the coding gain of the modified ECC circuit is improved by about 3 dB under the same BER of ${10^{-6}}$. The FPGA experimental results show that the RS ECC circuit has about an order of magnitude lower BER than the demodulation circuit when under the same coils distance. Therefore, the RS ECC circuit has more higher reliability of the communication in the system.

## Abbreviations

RS: Reed–Solomon
ECC: error correcting code
DAPSK: differential amplitude and phase shift keying
BER: bit error rate
DASK: differential amplitude shift keying
DPSK: differential phase shift keying
BPSK: binary phase shift keying
ASK: amplitude shift keying
Eb/No: bit signal to noise ratio (SNR)
